# Supplementary figures and images for: Uncovering the hidden yeast diversity in fermented coffee: Insights from a shotgun metagenomic approach
Source: PLoS One. 2026 Feb 19;21(2):e0332370. doi: 10.1371/journal.pone.0332370 (PMC12919822; doi:10.1371/journal.pone.0332370)

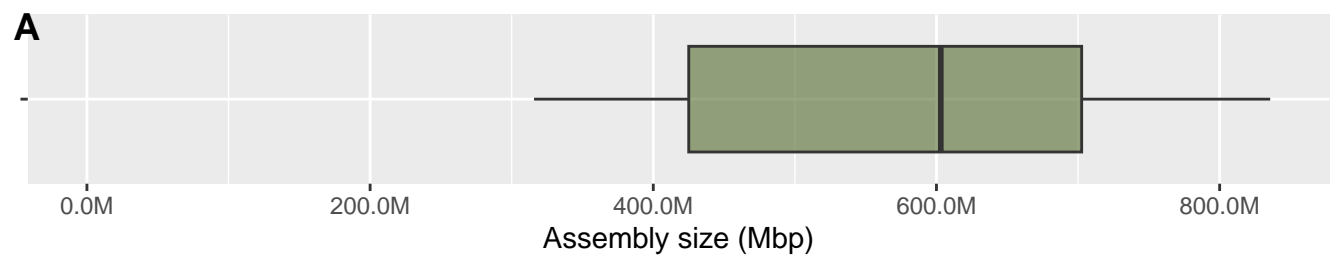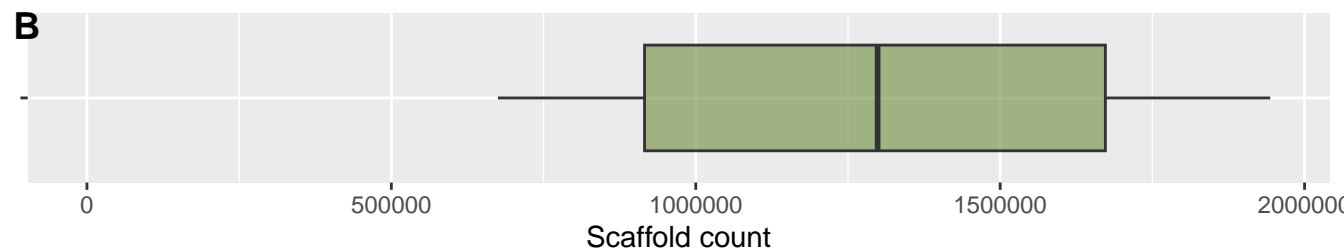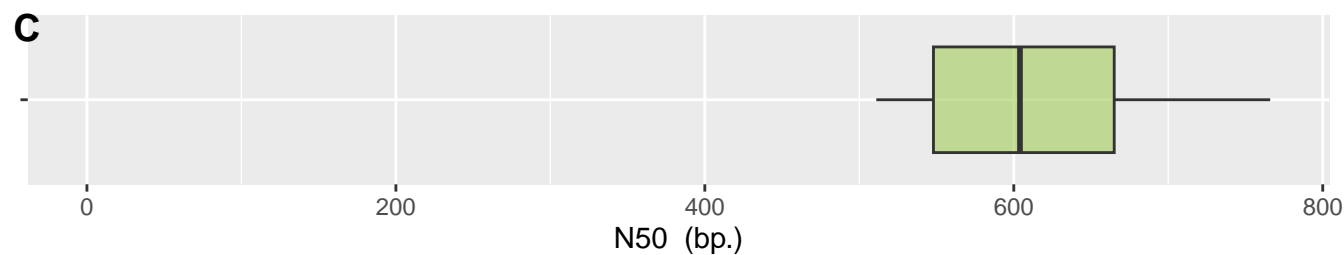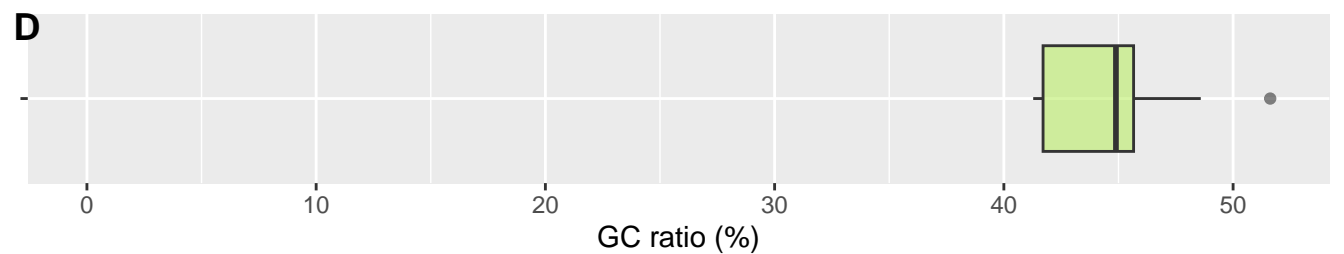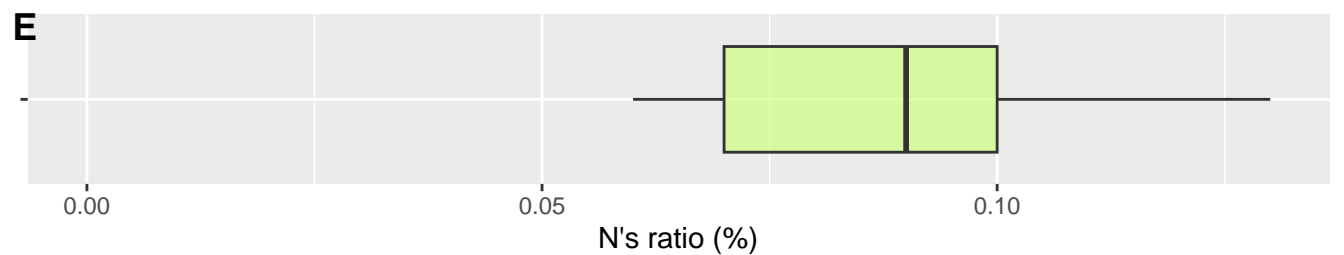

Supplement: S1 Fig — Each boxplot represents the distribution of five key assembly statistics across genomes reconstructed from nine metagenomic samples: (A) Total assembly size (Mbp), (B) Number of scaffolds per assembly, (C) N50 value (bp), (D) GC content (%), and (E) N base content as a percentage of total assembly. Boxes represent the interquartile range, the horizontal line indicates the median, and whiskers extend to 1.5 × the interquartile range. Assembly size is presented in megabase pairs (Mbp) using a scale transformation for readability. (PDF) [file pone.0332370.s001.pdf]

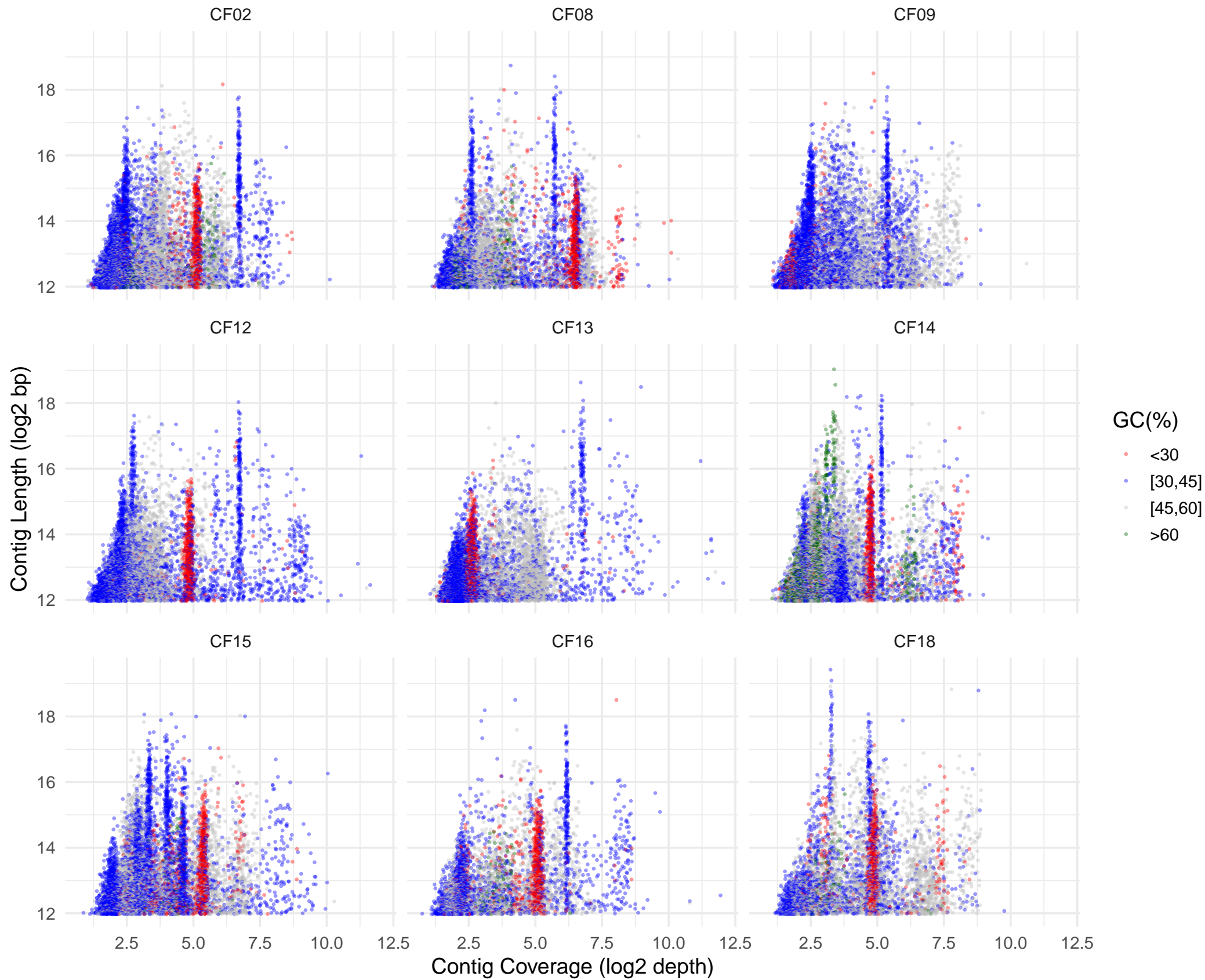

Supplement: S2 Fig — Each point represents a contig with length ≥4,000 bp. The X-axis corresponds to the log₂-transformed average sequencing depth, while the Y-axis shows the log₂-transformed contig length. Points are colored according to GC content binned into four categories: < 30% (red), [30–45%] (blue), [45–60%] (gray), and >60% (dark green). Only contigs with sufficient length were retained to reduce noise from fragmented sequences. Each panel corresponds to a different sample (CF02–CF18. (PDF) [file pone.0332370.s002.pdf]

Assembly Length (log2, M)

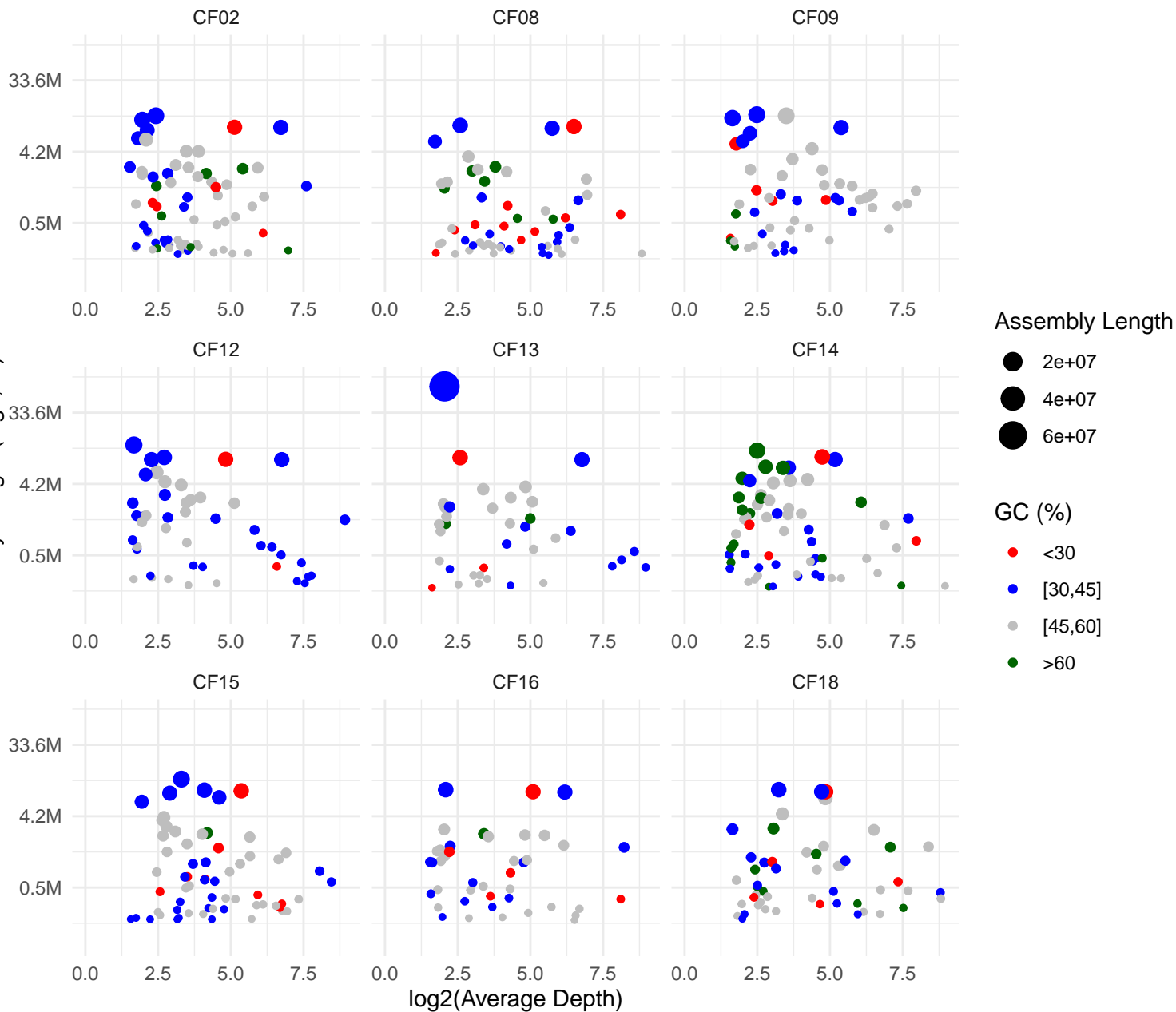

Supplement: S3 Fig — Each point represents a bin, plotted by its average sequencing depth (log₂-transformed, x-axis) and total assembly length (log₂-transformed, y-axis). Point size scales with assembly length. Points are colored according to GC content binned into four categories: < 30% (red), [30–45%] (blue), [45–60%] (gray), and >60% (dark green). Faceted panels display individual samples (CF02 to CF18. (PDF) [file pone.0332370.s003.pdf]

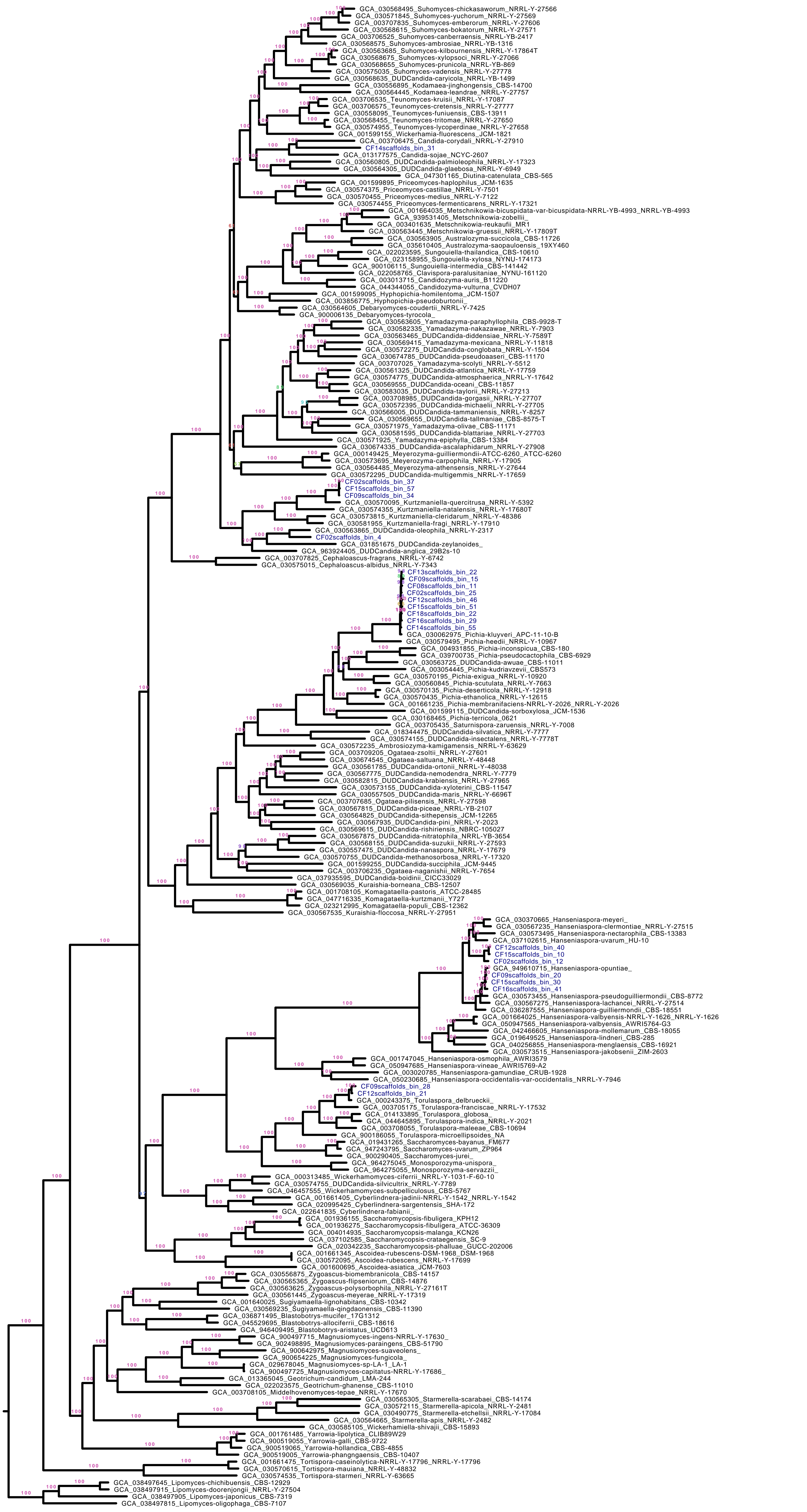

Supplement: S4 Fig — The tree was constructed from a concatenated alignment of 832 conserved single-copy protein-coding genes and includes reference genomes representing all major Saccharomycotina lineages. MAGs are shown in blue to distinguish them from reference genomes. Ultrafast bootstrap (UFB) support values are shown for relevant nodes. (PDF) [file pone.0332370.s004.pdf]
