# Supplementary material for: Uncovering the hidden yeast diversity in fermented coffee: Insights from a shotgun metagenomic approach
Source: PLoS One. 2026 Feb 19;21(2):e0332370. doi: 10.1371/journal.pone.0332370 (PMC12919822; doi:10.1371/journal.pone.0332370)
Supplement: Attachment — Submitted filename: Rebutal_letter_RevAida.docx [file pone.0332370.s010.docx]

PONE-D-25-47268

Uncovering the Hidden Yeast Diversity in Fermented Coffee: Insights from a Shotgun Metagenomic Approach

PLOS ONE

**Rebuttal Letter**

R:/ We sincerely thank the editor and reviewers for their time and effort in carefully evaluating our manuscript, and for their thoughtful comments, constructive critiques, and valuable recommendations. We are confident that these suggestions have substantially improved the quality and clarity of the manuscript. We have made every possible effort to address all comments and enhance the manuscript accordingly.

1. Please ensure that your manuscript meets PLOS ONE's style requirements, including those for file naming. The PLOS ONE style templates can be found at

<https://journals.plos.org/plosone/s/file?id=wjVg/PLOSOne_formatting_sample_main_body.pdf> and

<https://journals.plos.org/plosone/s/file?id=ba62/PLOSOne_formatting_sample_title_authors_affiliations.pdf>

R:/ We carefully reviewed the journal’s guidelines and revised the manuscript format accordingly.

2. In your Methods section, please provide additional information regarding the permits you obtained for the work. Please ensure you have included the full name of the authority that approved the field site access and, if no permits were required, a brief statement explaining why.

R:/ A detailed paragraph describing the authorization for sample collection has been added to the Methods section. This is the paragraph: “Sample collection was conducted under the valid permit issued by Resolution No. 1508 of September 6, 2018. The permit was granted by the NATIONAL ENVIRONMENTAL LICENSING AUTHORITY OF COLOMBIA (ANLA) to the Post-Harvest Coffee Research Group (COL0006634) in the implementation of the project POS101031, “Characterization of the microbial composition in the fermentation of coffee produced in the department of Quindío”; This was within the broader framework of the project “Experimental Development for the Competitiveness of the Coffee Sector of the Department of Quindío”; code 2017000100099, in agreements signed with the National Colombian Coffee Growers Federation (Cenicafé—Crossref Funder ID 100019597), No. 002-of-2020”.

3. Please note that PLOS One has specific guidelines on code sharing for submissions in which author-generated code underpins the findings in the manuscript. In these cases, we expect all author-generated code to be made available without restrictions upon publication of the work. Please review our guidelines at <https://journals.plos.org/plosone/s/materials-and-software-sharing#loc-sharing-code> and ensure that your code is shared in a way that follows best practice and facilitates reproducibility and reuse.

R:/ No proprietary or custom scripts were generated in this study. The analyses were conducted using publicly accessible tools, including IQ-TREE, MEGAN, BLAST, MetaBAT, BUSCO, and others. The commands used were described in supplementary material (S4 Software).

4. We note that the grant information you provided in the ‘Funding Information’ and ‘Financial Disclosure’ sections do not match. When you resubmit, please ensure that you provide the correct grant numbers for the awards you received for your study in the ‘Funding Information’ section.

R:/ The funding information was reviewed and updated to align with the Financial Disclosure section.

5. Thank you for stating the following financial disclosure:
“AEPM This research was developed under the project: Experimental Development for the Competitiveness of the Coffee Sector of the Department of Quindío, code 2017000100099, financed by the General System of Royalties, Gobernación de Quindío in agreements signed with the National Colombian Coffee Growers Federation (Cenicafé—Crossref Funder ID 100019597). No. 002-of-2020.” Please state what role the funders took in the study. If the funders had no role, please state: "The funders had no role in study design, data collection and analysis, decision to publish, or preparation of the manuscript." If this statement is not correct you must amend it as needed.
Please include this amended Role of Funder statement in your cover letter; we will change the online submission form on your behalf.

R:/ The text has been revised accordingly, and all funding-related information has been removed from the manuscript. In addition, the suggested sentence has been included in the submission system as a comment addressed to the editor.

6. Thank you for uploading your study's underlying data set. Unfortunately, the repository you have noted in your Data Availability statement does not qualify as an acceptable data repository according to PLOS's standards. At this time, please upload the minimal data set necessary to replicate your study's findings to a stable, public repository (such as figshare or Dryad) and provide us with the relevant URLs, DOIs, or accession numbers that may be used to access these data. For a list of recommended repositories and additional information on PLOS standards for data deposition, please see <https://journals.plos.org/plosone/s/recommended-repositories>.

R:/ The Data Availability information included in the Methods section has been revised to comply with the journal’s guidelines. The sentence “The raw data can be accessed through the following link: https://www.ncbi.nlm.nih.gov/sra/PRJNA1305218. The sample identifiers are SRR34971509, SRR34971508, SRR34971516, SRR34971515, SRR34971514, SRR34971513, SRR34971512, SRR34971511, and SRR34971510” was added.

7.  Please include captions for your Supporting Information files at the end of your manuscript, and update any in-text citations to match accordingly. Please see our Supporting Information guidelines for more information: http://journals.plos.org/plosone/s/supporting-information.

R:/ Captions for supplementary Figure 1 and supplementary tables were added to the manuscript. This caption was added in the end of the manuscript.

**“**S1 Table. Summary of sample conditions and associated physical parameters.

S2 Table. Accession numbers of yeast genomes included in the phylogenomic analysis.

S3 Table. Taxonomic classification and quality metrics of yeast metagenome-assembled genomes (MAGs) derived from coffee fermentation metagenomes.”.

**Comments to the Author**

5. Review Comments to the Author

Reviewer #1: The paper entitled “Uncovering the Hidden Yeast Diversity in Fermented Coffee: Insights from a Shotgun Metagenomic Approach” by Katherine Bedoya-Urrego, Aida Esther Peñuela-Martínez, and Juan Fernando Alzate is an original descriptive study of fungal diversity in coffee fermentations. The paper is well-written, thoroughly analyzed, and clearly presented.
Although there is a long tradition of studies focusing on the culturable fraction of fungi and metabarcoding of molecular marker DNA (ITS, ribosomal RNA genes) associated with various coffee bean fermentations, the metagenomic approach—understood as the sequencing of all genes and species present in a sample or habitat—represents a novel perspective in this field. The article is original and interesting; however, in its current version, the identification of yeasts based solely on metagenomic information is insufficient for publication in PLOS ONE (IF 2.6, Q1–Q2). The arguments supporting this opinion are presented below. If the authors are able to enlarge the genomic and metagenomic analyses, the paper could be reconsidered. In my opinion, the study would have been strengthened by including genomes from the cultivable fungal fraction. This information could help confirm the relative abundance of each species, improve species-level identifications, and enable the investigation of important genes related to relevant phenotypic traits during mixed coffee fermentation, among other opportunities.
I understand that there may be limitations in achieving species-level identification; however, in some cases, species belonging to certain clades are not mentioned. In addition to phylogenomic analyses, species-level identification should be further supported by ANI and AAI estimates, in silico DNA–DNA hybridization, and other complementary analyses. If the authors consider it appropriate and the results suggest the discovery of a potential new species, they could mention this possibility without committing to a formal description.

R:/ We thank the reviewer for this valuable comment and fully agree that integrating genomes from cultivated isolates could further enrich our understanding of the yeast community in coffee fermentation. However, we would like to emphasize that MAGs provide unique and complementary insights that are not attainable through culture-based approaches alone. First, MAGs capture the genomic diversity of yeast taxa that often escape isolation using traditional methods. Many yeasts involved in spontaneous fermentations, especially those from the early stages or transient populations, are difficult to recover in pure culture due to fastidious growth requirements, competition, or dependence on specific microbial interactions. Thus, the metagenomic framework allows the recovery and genomic characterization of previously unrecognized or novel lineages, as evidenced in our study by MAGs that did not match any currently described species. Second, MAGs enable a genome-resolved view of the active community structure, providing more accurate estimates of relative abundance within the fermentation matrix. This avoids cultivation bias and reflects the actual *in-situ* representation of each yeast species in the process.

While cultivation-based approaches remain invaluable for experimental validation, metagenomics offers an ecologically relevant, culture-independent perspective that broadens our understanding of microbial participation in complex fermentations like coffee. We view our MAG-based analysis as a complementary and foundational framework upon which future studies integrating cultivable isolates and phenotypic analyses can build.

We fully agree with the reviewer on the importance of confirming taxonomic assignments using the most current and scientifically supported approaches. For this reason, we selected a phylogenomic strategy, which is considered the gold standard for taxonomic classification, as it provides a robust framework for accurate evolutionary inference based on orthologous loci across genomes and topological context. ANI is other well supported strategy for taxonomic assignment; however, ANI thresholds and tools are primarily optimized for prokaryotic genomes, and their applicability to yeast species remains limited and not yet standardized. For these reasons, we prioritized a phylogenomic approach.

Beyond taxonomic analyses, the study does not fully exploit the large amount of data generated to perform comparative genomic analyses among the identified yeast species. Such analyses could provide insights into the role of widely distributed yeast species in coffee bean fermentation. Furthermore, no genes or metabolic pathways relevant to fermentation—such as extracellular enzymes, carbohydrate assimilation, secondary metabolites, fermentation metabolism, and other functional aspects—are highlighted.

R:/ Metabolic analyses were incorporated into the Results and Discussion sections. Comparative analyses of the metabolic potential among the yeast MAGs were conducted to identify functional and genomic distinctions between the detected species.

If the metagenomic work is intended to remain descriptive, it should also include bacterial and archaeal communities or microbiomes. It would also be valuable to compare the presence or absence of fungal genera detected by similar or different approaches in previous coffee fermentation studies with those identified in this work.

R:/ We thank the reviewer for this valuable comment. We fully agree that including bacterial and archaeal communities would provide a more comprehensive view of the coffee fermentation microbiome. However, our study was specifically designed to address a current knowledge gap regarding the yeast profile in coffee fermentation, using a metagenomic and high-resolution taxonomic approach. The bacterial component represents a highly complex system that extends beyond the scope of this work and would require a dedicated and independent analysis. We believe that focusing on the yeast fraction allows us to provide novel and detailed insights into their metabolic potential and ecological role during coffee fermentation.

We appreciate the reviewer’s insightful suggestion of compare our results with other studies in Colombia. In response, we have included in the Discussion a comparison with local studies that evaluated yeast profiles in fermented coffee samples using a metataxonomic approach. This addition allows us to contextualize our metagenomic findings with previously reported fungal community compositions obtained through different methodologies.

In Figure 4, sequences corresponding to several animals such as Dendroctonus, Caenorhabditis, Callosobruchus, and probably Drosophila are reported. This likely reflects an issue with inadequate data filtering. Please provide an explanation. For instance, Dendroctonus is a conifer bark beetle.

R:/ We thank the reviewer for this valuable observation, as we agree that it is important to carefully consider potential false positives in metagenomic-based studies. We would like to clarify that this work is based on shotgun metagenomic sequencing, in which DNA from all organisms present in the sample—including non-target eukaryotes—can be detected. Given that the coffee fermentation on farms is performed under artisanal and open conditions, it is expected that reads from environmental organisms such as nematodes and arthropods may appear in the metagenomic dataset.

Therefore, the MEGAN assignments should not be interpreted as definitive taxonomic identifications but rather as a broad overview of the taxonomic composition of the sample. The detection of taxa such as *Drosophila*, *Caenorhabditis*, or *Dendroctonus* does not imply their active participation in the fermentation process but instead could reflects the presence of environmental or incidental invertebrate DNA. Our intention in presenting the MEGAN results was to provide a comprehensive view of the total DNA diversity within the fermentation environment.

However, since the focus of this study is on yeasts, we validated and refined their taxonomic assignments using phylogenomics, which currently represents the gold standard for high-resolution and accurate classification of microbial genomes, including MAGs.

I consider that Figures 1, 2, 3, and 9 should be moved to the supplementary material section, as they only present quality analyses of massive sequencing.

R:/ Figures 1,2,3 were moved to supplementary material according to the reviewer suggestion. However, we considered that the Figure 9 is relevant for the results since it represents the taxonomy and relative abundance of MAGs that is an important axis of this work. For this reason, we would like to retain the figure within the Results section

Regarding taxonomic assignments (Fig. 4): Taxonomic assignment using MEGA alone is quite limited, as it relies on outdated databases. It is therefore recommended to use more updated and robust tools such as QIIME2 (<https://library.qiime2.org/quickstart/moshpit>).

R:/ We agree with this assessment; accurate taxonomic assignment is essential for this type of analysis. The alignment-based taxonomic methods (such as MEGAN, QIIME, or other similarity-based tools) are inherently limited by database completeness and sequence homology thresholds, which can result in both false-positive and false-negative identifications. For this reason, these methods were used only as a preliminary exploratory step, whereas the final classification of yeast MAGs was established through robust phylogenomic reconstruction.

Minor details: Line 114: Change −20 °C to −20°C (no space between the number and unit). Lines 722–723: Debaryomycetaceae sp. — the term “sp.” is unnecessary.

R:/ Corrections were made in the manuscript.

Finally, regarding omics approaches to explore the coffee fermentation microecosystem and its effects on cup quality, I believe that some important works that could strengthen the research’s international context are missing from the introduction or discussion.

R:/ Thanks to the reviewer for this helpful observation. In response, we have added a paragraph to the Introduction to address this point.

“Metagenomics is a powerful approach for exploring yeast diversity in food fermentation processes, providing an integrated and less biased view of this taxonomic group and their functional ecology [13,14]. Previous metagenomic studies have revealed dynamic changes in gene repertoires and temporal shifts in both bacterial and yeast communities during coffee fermentation. These variations, influenced by geographical origin and fermentation type, have been shown to markedly affect the metabolic potential of the microbiota and, consequently, the sensory quality of coffee [15]. Metagenomic binning enabled the reconstruction of metagenome-assembled genomes (MAGs), allowing a more precise assessment of yeast contributions to the fermentation process and providing high-resolution taxonomic classification [16,17].”

Reviewer #2: Summary

Bedoya-Urrego et al. apply shotgun metagenomics, binning, and a phylogenomic framework based on a large set of single-copy proteins to profile Saccharomycotina yeasts in spontaneous Colombian coffee fermentations. The abstract reports 24 yeast MAGs (later 22 after filtering), dominated by Pichia kluyveri, with additional Hanseniaspora, Torulaspora, and Kurtzmaniella lineages and putative novel taxa. The study is timely and relevant; however, key reporting gaps (eukaryotic MAG quality control), internal inconsistencies (counts; assembly strategy), and methodological ambiguities prevent full evaluation.

R:/ We thank the reviewer for identifying these issues. We have carefully revised the manuscript to resolve the inconsistencies and improve clarity. We acknowledge that the initial version contained inconsistencies in the total number of yeast MAGs reported. After applying our filtering criteria (completeness, contamination, and phylogenomic quality), the final dataset includes 22 high-quality yeast MAGs. This value has now been reviewed, reconciled, and made consistent across the Abstract, Results, Methods, and Discussion. Additionally, we have expanded the Methods section to explicitly describe the quality-control procedures applied to eukaryotic MAGs and incorporated new analyses to improve the clarity and accuracy of the results in the revised manuscript.

Major revisions

1. Eukaryotic MAG quality control not reported
The manuscript does not present per-MAG completeness/duplication (e.g., BUSCO) or contamination estimates (e.g., EukCC), nor a MAG-level QC table. This is core to genome-resolved metagenomics. Please report for each MAG: BUSCO lineage/version with C/F/M/D %, an explicit contamination estimate (e.g., EukCC), size, N50, scaffold count, GC%, median depth, and breadth.

R:/ Thank you for this valuable observation. We assessed genome completeness duplication, Missing and fragmentation using the BUSCO software. These values have been added to Supplementary Table 3. In BUSCO (v.5.2.2), we used the saccharomycetes_odb10 database and Candida albicans as the reference strain.

Other Bin/MAG metrics such as, size, N50, scaffold count and average depth are described in the S3 Table. The breadth genome was not calculated in this study because as far as we understand, this metric is calculated by mapping reads to a well-defined reference genome. Because the genomes analyzed in this study correspond to de novo assembled yeast MAGs recovered from metagenomic data, and no appropriate reference genomes exist, this metric is not applicable in our context and may lead to misleading interpretations. Instead, following, we assessed completeness and duplication using BUSCO for MAG quality evaluation

2. Workflow clarity: per-sample assemblies vs. co-assembly
Results describe one assembly per sample; Methods describe mapping to co-assembled scaffolds. These are presented as a single workflow though they are distinct.
Please state unambiguously which assembly underlies binning, coverage, and figures (per-sample, co-assembly, or both) and align text/metrics accordingly.

R:/ Thank you for this observation. We acknowledge that there was an error in the description of the binning workflow in the methodology section. The sentence has been corrected
“Sequencing depth for each MAG was estimated by mapping quality-filtered reads to the corresponding binned scaffolds generated by metaSPAdes, using Bowtie2 (v2.3.4.3) with default alignment parameters ”

3. Counts and tallies disagree
Abstract: 24 MAGs; species-level tallies sum to 22; one sentence mentions removal of a single MAG (would total 23).
Please reconcile counts across Abstract, Results, legends, and supplements.

R:/ We selected 22 MAGs after applying the filtering parameters. This value was reviewed and reconciled throughout the manuscript.

4. Read handling for MEGAN is misdescribed
Reads are said to be “merged into longer single-end reads using “seqtk”,” which does not generate longer consensus reads. The manuscript also alternates between MEGAN on reads vs. on scaffolds.
Please correct the tool description and specify whether MEGAN used reads or scaffolds, which classifier/database (e.g., DIAMOND + NR; version/date), and LCA parameters.

R:/ We thank the reviewer for pointing out the inconsistencies regarding read handling and MEGAN input. We have now corrected the description of the workflow and clarified the exact data type used for MEGAN-based taxonomic assignment. Taxonomic analysis in MEGAN was performed using the extended reads generated after merging paired-end sequences with FLASH v1.2.11 (Fast Length Adjustment of Short Reads). Paired-end reads were processed with FLASH using default parameters to merge overlapping read pairs. The resulting merged reads were then aligned against the reference database using DIAMOND, and the resulting alignments were imported into MEGAN for taxonomic assignment based on the LCA algorithm. The DIAMOND version and the specific LCA parameters used in MEGAN have been added to the Methods section of the manuscript as follows:

“Metagenomic taxonomic assignment was performed using MEGAN (v.6.25) [20] to characterize the microbial composition of the coffee fermentation samples. Raw paired-end reads were first quality trimmed with *cutadapt* to remove adapters and low-quality bases. Subsequently, forward and reverse reads were merged into longer single-end reads using FLASH tool (v1.2.11) [21], thereby improving taxonomic resolution by providing longer sequence context. The merged high-quality reads were then aligned with DIAMOND (v2.0.14.152) tool and DAA files were imported into MEGAN for taxonomic assignment based on the lowest common ancestor (LCA) algorithm (default parameters: Min Score: 50, Top Percent: 10%, and Max Expected: 0,001)”

5. Abundance is visualized but not quantified
A length-vs-depth scatterplot is presented as “relative abundance,” but no explicit metric or presence threshold is provided.
Please provide a per-sample, per-MAG abundance table (e.g., reads mapped per kb; TPM/RPKM) and breadth thresholds for presence.

R:/ To address this important comment, we calculated TPM values for all MAGs/BINs and have now included these data in Supplementary Table 4. In addition, we retained only those BINs that recovered at least 50% of the expected genome size of the corresponding reference strain for each identified species.

6. Host (Coffea) depletion undocumented
Plant sequences are acknowledged; no reference genome, mapping criteria, or % removed are reported.
Please specify the Coffea reference(s), filtering criteria, and per-sample host removal summary.

R:/ In our workflow, host (Coffea) depletion was not performed prior to assembly. Instead, after generating the metagenome assembly, we applied a binning approach, which groups assembled contigs into genome bins based on sequence composition and differential coverage patterns. From these bins, we selected those corresponding to the dominant yeasts in the metagenomes. To ensure accurate taxonomic assignment, we additionally performed nucleotide BLAST searches to retain only contigs matching yeast sequences, and these curated bins were subsequently validated as high-confidence yeast MAGs.

7. Phylogenomic model scope vs. claims
Deep-rank statements (e.g., family non-monophyly) are paired with uniformly high UFB support under a single model description.
Please either provide a brief robustness check (partitioning and/or site-heterogeneous model) or temper claims; indicate whether key placements are model-stable.

R:/ The statement referring to “possible non-monophyly” was removed from the Results section, as we agree that it could lead to misinterpretation. The LG4M model selected in this study does not use partitions; instead, it is an empirical amino acid substitution model that accounts for rate heterogeneity across sites in protein alignments.

Methodological clarifications

BLASTN thresholds. “E-value = 0” and “bitscore > 10,000” are not biologically interpretable cutoffs. Define alignment length, % identity, and coverage criteria; indicate whether reciprocal hits or AAI/ortholog identity supported species calls.
Rank terminology. The text refers to “class-level” structure while listing “-ales” groups (orders). Harmonize rank labels with current taxonomy.
Software versions/commands. Resolve the Cutadapt version mismatch (“version 3” vs “v2.10” in parameters). Provide exact versions and key command lines (trimming, assembly, binning, mapping, phylogeny) in Supplementary Methods.

R:/ We appreciate the reviewer’s comment regarding the use of stringent BLASTN cutoffs. We agree that cutoffs such as E-value = 0 and bitscore > 10,000 are not themselves biological thresholds, but rather computational stringency filters. Our intention was not to imply that these values have intrinsic biological meaning, but to avoid false-positive recruitment of non-yeast sequences and ensure that only very high-confidence matches were retained when assigning contigs to yeast genomes. This is because an E-value of 0 ensures that the probability of the match occurring by chance is below machine floating-point precision. And a Bitscore > 10,000 corresponds to very long alignments.

The species were supported with phylogenomic approach that provides a comprehensive, model-based method that integrates hundreds of conserved orthologs and is widely accepted as the most accurate tool for taxonomic classification of yeast genomes and MAGs.

The most important software used in this research, such as CUTADAPT, METASPADES, BOWTIE, and METABAT were described in supplementary material (S4).

Other

Flavor/functional claims. Several passages imply causal impacts on sensory outcomes; no metabolomics/sensory data are presented. Either add minimal genomic context (e.g., presence/absence of ester/pectinase loci per MAG) or consistently frame these as literature-based hypotheses.

R:/ We performed a functional prediction analysis for the dominant yeast genera detected in the fermented coffee samples *Kurtzmaniella, Torulaspora, Hanseniaspora*, and *Pichia*. The analysis is described in detail in the Results section and further interpreted in the Discussion.

“Megan sequencing depth” → mean sequencing depth.
“CF15 showed two distinct species of Hanseniaspora.”
Consistent capitalization (MEGAN), consistent “phylogenomics” wording, report “UFBoot support = 100%”.
Avoid mixing CFxx and Fxx for the same batches; provide a simple crosswalk table.

R:/ Thank you for the valuable corrections. All of them have been incorporated into the new version of the manuscript.
