# Supplementary material for: Uncovering the hidden yeast diversity in fermented coffee: Insights from a shotgun metagenomic approach
Source: PLoS One. 2026 Feb 19;21(2):e0332370. doi: 10.1371/journal.pone.0332370 (PMC12919822; doi:10.1371/journal.pone.0332370)
Supplement: Attachment — Submitted filename: rebuttal letter_rev_kathe.docx [file pone.0332370.s011.docx]

PONE-D-25-47268R1

Uncovering the Hidden Yeast Diversity in Fermented Coffee: Insights from a Shotgun Metagenomic Approach

PLOS One

**Rebuttal Letter**

We sincerely thank the editor and reviewers for their constructive comments and valuable suggestions, which have significantly improved the quality and clarity of the manuscript. We have addressed all comments accordingly.

(i) Please add an explicit MAG contamination estimate (e.g., EukCC or comparable) and consider reporting read-mapping breadth to each MAG (breadth can be calculated against the MAG scaffolds themselves, not only against external references)

R/: Thanks for the recommendation. We perform the analysis with the EukCC and report the contamination and completeness in the S3 table (column R and Q). The breath of coverage (%) of each MAG also was reported (column P).

(ii) Resolve remaining reproducibility inconsistencies (notably the Cutadapt version mismatch) and ensure database/version/date details are complete.

R/: Cutadapt version was revised, and the correction was made in Material and Methods section

(iii) Revise the BLAST screening description to include interpretable criteria (alignment length, % identity, and coverage), even if phylogenomics is the primary basis for species assignment

R/: The BLAST filtering parameters (E-value = 0 and bit score ≥ 10,000) resulted in alignment blocks of at least 2,000 bp. We verified that the minimum sequence identity of these aligned blocks relative to their respective reference genomes was 70%. For the phylogenomic analysis, single-copy protein-coding genes were predicted using AUGUSTUS, and orthology inference was performed with SonicParanoid, as described in the Methods section. This annotation and orthology framework provides robust support for the phylogenomic analyses presented in this study.

(iv) (iv) harmonize taxonomic rank terminology and ensure any residual “non-monophyly” language is tempered unless supported by robustness checks. With these minor clarifications, I would support publication.
(v) R/: The sentence has been removed from the results section.
